# Using non-invasive behavioral and physiological data to measure biological age in wild baboons

**DOI:** 10.1007/s11357-024-01157-5

**Published:** 2024-05-02

**Authors:** Chelsea J. Weibel, Mauna R. Dasari, David A. Jansen, Laurence R. Gesquiere, Raphael S. Mututua, J. Kinyua Warutere, Long’ida I. Siodi, Susan C. Alberts, Jenny Tung, Elizabeth A. Archie

**Affiliations:** 1 Department of Biological Sciences, University of Notre Dame, Notre Dame, IN USA; 2 Department of Biology, Duke University, Durham, NC USA; 3 Amboseli Baboon Research Project, Amboseli National Park, Kajiado, Kenya; 4 Department of Evolutionary Anthropology, Duke University, Durham, NC USA; 5 Duke University Population Research Institute, Duke University, Durham, NC USA; 6 Department of Primate Behavior and Evolution, Max Planck Institute for Evolutionary Anthropology, 04103 Leipzig, Germany; 7 Canadian Institute for Advanced Research, Toronto, M5G 1M1 Canada; 8 Faculty of Life Sciences, Institute of Biology, Leipzig University, Leipzig, Germany

**Keywords:** Social determinants of health, Biological aging clock, Non-human primate, Animal model of aging, Longitudinal research

## Abstract

Biological aging is near-ubiquitous in the animal kingdom, but its timing and pace vary between individuals and over lifespans. Prospective, individual-based studies of wild animals—especially non-human primates—help identify the social and environmental drivers of this variation by indicating the conditions and exposure windows that affect aging processes. However, measuring individual biological age in wild primates is challenging because several of the most promising methods require invasive sampling. Here, we leverage observational data on behavior and physiology, collected non-invasively from 319 wild female baboons across 2402 female-years of study, to develop a composite predictor of age: the non-invasive physiology and behavior (NPB) clock. We found that age predictions from the NPB clock explained 51% of the variation in females’ known ages. Further, deviations from the clock’s age predictions predicted female survival: females predicted to be older than their known ages had higher adult mortality. Finally, females who experienced harsh early-life conditions were predicted to be about 6 months older than those who grew up in more benign conditions. While the relationship between early adversity and NPB age is noisy, this estimate translates to a predicted 2–3 year reduction in mean adult lifespan in our model. A constraint of our clock is that it is tailored to data collection approaches implemented in our study population. However, many of the clock’s components have analogs in other populations, suggesting that non-invasive data can provide broadly applicable insight into heterogeneity in biological age in natural populations.

**Supplementary Information:**

The online version contains supplementary material available at 10.1007/s11357-024-01157-5.

## Introduction

For most species, aging is an inevitable biological process. It is reflected in declining cellular, tissue, and organ-level function with age, leading to an increased risk of disease and death [1, 2]. These processes exhibit considerable heterogeneity, with some individuals aging in different ways and at different rates than others [3, 4]. Measuring this variation and understanding its causes and consequences is essential to learn how evolution has shaped the aging process and to improve human health across the life course.

Recently, this line of research has been enriched by animal models studied in their natural habitats, where they experience species-typical environments and stressors (e.g., [5–7]). Such models have helped place human aging in an evolutionary context [8–16] and have shed light on the social and environmental drivers of aging [17–19]. The particular value of natural animal populations lies in their ability to provide prospective, longitudinal data on the major events and social conditions of individuals’ lives, from birth to death, allowing researchers to pinpoint the conditions and exposure windows that most affect aging (e.g., [20–22]). Such studies have uncovered striking effects of early-life circumstances on adult health and mortality and tested the relative importance of early-life vs adult conditions in predicting individual heterogeneity in biological age (e.g., [20, 23–25]).

However, while natural animal populations may illuminate drivers of biological aging, tools to measure biological age in these systems lag behind those for humans and laboratory animals [26, 27]. Ideally, such tools should reflect heterogeneity in biological age and predict adverse aging-related outcomes, including all-cause mortality. One set of tools that meets these criteria in humans and some animals are epigenetic or biomarker-based clocks (e.g., [3, 28, 29]). These “aging clocks” apply multidimensional data within predictive machine learning frameworks to predict an individual’s known chronological age, mortality risk, or pace of aging; for clocks calibrated to predict chronological age, deviations from these predictions are often used to infer individual variation in biological age [28–30]. However, the source material for these clocks—typically blood or other tissue samples—can be difficult to collect in free-living animals. The need for invasive sample collection also constrains the ability to measure biological age in repeated samples across the life course (a requirement for researchers who want to measure heterogeneity in biological age within individual lifespans [31]).

One alternative is to develop clocks that leverage data that can be collected without invasive sampling (e.g., behavioral data, biomarkers from animal feces or urine). Such a clock would share some similarities with visual assessments of age (e.g., [32, 33]) and frailty indices developed for humans, which center on physical functioning and the ability to perform activities of daily living (reviewed in [34]). Such indices remain among the most powerful measures of human mortality risk developed to date [35–38]. To our knowledge, no non-invasive age-predicting clocks have been built for non-human primates. However, frailty indices have been developed for primates and rodent models, and some have been used to create clocks that predict chronological age and life expectancy (although they often incorporate invasive measures: [26, 39–42]).

Here, we use supervised machine learning to create a composite age predictor in wild female baboons: the “non-invasive physiology and behavior age-predicting clock” (the NPB clock), based solely on data and samples that were collected non-invasively. To do so, we use longitudinal behavioral, demographic, and physiological data collected by the Amboseli Baboon Research Project (ABRP)—a 52-year longitudinal study of baboons in the Amboseli ecosystem in Kenya [43]. Baboons are highly social, terrestrial primates that experience a wide range of natural social and ecological stressors. Like humans, adult mortality in baboons is strongly predicted by harsh early-life conditions and social isolation in adulthood [44, 45]. Unlike many human population studies, these and other potential socio-environmental predictors of biological age can be measured prospectively, in real time, and longitudinally across the life course [46].

Before building our age-predicting clock, we began by testing for age associations in 78 non-invasively measured traits from the ABRP’s long-term monitoring data. We then used 49 of these traits (those that were age-associated) to construct the NPB age-predicting clock by evaluating the performance of three machine learning methods. Next, we used age predictions from the NPB clock to test whether (i) individuals whose age predictions are older than their known ages exhibit higher mortality than those who are predicted to be young-for-age and (ii) socio-environmental conditions in early life and adulthood, including early-life adversity, are linked to old-for-age clock predictions in adulthood [45, 47, 48]. We found that variation in NPB clock predictions is positively correlated with mortality risk, and early-life adversity is linked to old-for-age NPB clock age predictions. Finally, we discuss our results as they relate to other age-predicting clocks for the Amboseli population, other animal models, and humans.

## Methods

### Study subjects and longitudinal data on female traits

Our subjects were 319 wild adult female baboons living in 14 distinct social groups studied by the Amboseli Baboon Research Project from September 1975 to September 2021 (ABRP; [43]). These groups were the product of group fissions and fusions from two original groups, first studied in 1971 and 1981 (no more than six groups were studied at any given time). Members of this population are hybrids between yellow and anubis baboons (*Papio cynocephalus* and *P. anubis*), with majority yellow baboon ancestry [49–51]. We focused on female baboons because male postnatal dispersal creates observation gaps for males across adulthood. Female age was known to within a few days’ error for 295 of the females in our data set, and for the remaining 24 females (7.5%), age was known to within 3 months’ error. All subjects were > 4 years old, a cutoff that corresponds to approximately the earliest age of female sexual maturity in this population (median age at menarche in Amboseli = 4.5 years [52]).

All ABRP study animals are visually identifiable by trained observers who monitor each study group two to four times per week, year-round. During each monitoring visit, observers collect many different types of data, including information on individual activity budgets, social interactions, offspring care, illnesses and injuries, and reproductive states [53]. They also collect non-invasive fecal samples from known animals, which are used to quantify individual parasite loads and steroid hormone levels. For most subjects (256 of 319 females), we also had data on six early-life conditions that cumulatively, and in some cases individually, predict mortality in this population [45]: (1) maternal death before 4 years of age; (2) the presence of a close-in-age younger sibling, which may divert maternal resources; (3) drought in the first year of life; (4) maternal social isolation in the first 2 years of life; (5) low maternal social dominance rank at birth; and (6) large group size at birth, which is linked to elevated resource competition [45]. See the Supplementary Information for details on how these variables were measured.

ABRP research is approved by the Institutional Animal Care and Use Committees at Duke University and the University of Notre Dame and the Ethics Council of the Max Planck Society and adheres to the laws and guidelines of the United States, Germany, and Kenya.

### Testing which traits change with female age

Before building a composite age-predicting clock, we first identified 78 individual traits from the ABRP’s long-term monitoring data that could plausibly change with female age. We then tested each trait’s linear and quadratic (i.e., curvilinear) relationships with female age. Data on these 78 traits were collected by the ABRP between 1975 and 2021 for a range of 7 to 46 years (some data sets started after 1975 and one ended before 2021).

We grouped the 78 traits into ten categories (Table S1). Category 1 (activity budget, 18 traits) included the percentage of time females spent in basic activities in a given year of life (birthday to birthday), including resting, walking, feeding, grooming, or being groomed. We also considered the percentage of time females were observed with no groupmates in close spatial proximity, partitioned depending on whether they had a dependent infant, as having an infant is an important determinant of female activity and neighbors. Category 2 (endocrine measures, 4 traits) focused on concentrations of hormone metabolites in individual fecal samples. Category 3 (illness and injury, 7 traits) included the incidence of observable cases of illnesses and injuries in a given year of life. Category 4 (parasites, 2 traits) measured parasite burdens in individual fecal samples. Category 5 (reproduction, 5 traits) focused on reproductive variables, such as the duration of reproductive phases (e.g., cycling, pregnancy, postpartum amenorrhea) in days and the incidences of fetal loss and offspring survival in a given year of life. Category 6 (maternal care, 7 traits) included measures of the quality of care the focal females provided to their infants, contingent on having an infant, as assessed based on the percentage of time spent carrying, grooming, and nursing infants in a given year of life. Category 7 (social integration, 18 traits) included aggregate measures of social integration with adult males and other adult females in a given year of life. Category 8 (dyadic sociality, 4 traits) captured the strength and reciprocity of dyadic social bonds with adult males and other adult females in a given year of life. Category 9 (agonism, 5 traits) included measures of a female’s relative number of agonistic interactions in a given year of life, compared to other females living in the population at the same time. Finally, Category 10 (social dominance rank, 8 traits) included several measures of social dominance rank and change across adulthood. See Table S1 for a list and description of all the traits in each category and the Supplementary Information for details on how these traits were measured.

We began by testing which of the 78 traits exhibited linear or curvilinear relationships with female age using a two-part modeling strategy. First, before modeling our primary variable of interest (female age), we built initial models to determine which additional covariates should be included in models of each of the 78 traits. These initial models included all fixed and random effects known or suspected to explain variation in the trait from previous analyses in the Amboseli population. When no prior analyses of the trait had been conducted, we modeled the same variables used for the most similar trait (see Table S2 for definitions of the variables). The best-fitting fixed and random effects for these initial models were identified using the *dredge* function in the MuMIn package [54]. We chose models that minimized AIC; if multiple models had AIC values within two units, we selected the model with the lowest degrees of freedom (see Table S2). Second, we added linear or quadratic terms for female age to the best-supported initial model and tested if either or both of these terms were statistically significant (alpha = 0.05). Together, these two modeling steps revealed 49 traits that had significant linear or quadratic associations with female age (at alpha ≤ 0.05). All statistical analyses were conducted in R, version 4.0.3 [55, 56].

Most of the 78 traits were continuous variables (*N* = 66), while a minority were categorical variables with either two (*N* = 7) or three levels (*N* = 5). The 66 continuous traits were centered, standardized, and modeled using Gaussian error distributions using the *lmer* function from the lme4 package in R [57]. For categorical traits with two levels (*N* = 7), we created a generalized linear mixed model with a binomial error distribution using the *glmer* function also from lme4. For categorical variables with three levels (*N* = 5, including active rank change, rank relative to daughters, rank relative to mother, pregnancy outcome, and offspring survival), we created a multinomial model using the *brm* function from the brms package [58]. See the column labeled “model type” in Table S3 for correspondence between each trait and model type.

### Preparing data for the non-invasive physiology and behavior (NPB) clock

Following Schultz et al. [26], we used the 49 traits that exhibited significant linear or curvilinear associations with age to construct a supervised machine learning-based age predictor, the NPB clock. Sub-setting to age-associated traits is not a requirement of creating an age-predicting clock, but this form of feature selection is common [26, 30] and pre-selecting variables reduced the number of traits for which we had to impute missing data (described below). Most of our traits were measured annually for each female in each year of life (e.g., all traits under activity budgets, illness and injury, maternal care, social integration, dyadic sociality, agonism, and social dominance rank, and some traits under reproduction). For hormone and parasite measures, which were measured on the level of individual samples, we extracted the residual values for each sample correcting for key covariates (described above) and averaged these residuals within a given year of life. For the remaining female reproductive traits (e.g., the duration of pregnancy or ovarian cycling), we used female age at the start of the year in which the trait was measured.

The final data frame contained data on the 49 traits for 319 females across 2402 years of adult life (Fig. S1; mean = 7.53 years of observations per female; female chronological age ranged from 4 to 27 years). This data set excluded female-years in which the female was observed for < 60 days and female-years in which data on ≥ 65% of the traits (i.e., ≥ 32 of the 49 traits) were missing (Table S4). As a result, 26% of the cells in this data set were missing an observed value and required imputation (Fig. S1; note that a more conservative threshold of missingness—where years with ≥ 36% of traits missing [i.e., ≥ 18 of 49] were excluded—had little effect on clock age predictions; Fig. S2). Imputation occurred in two phases. First, if data were missing for a trait in a given year of life, for a given female, but information for that trait was available for that female in either the year immediately before or after, we extrapolated the observed value to the missing year. If information was available for both the year immediately before and after the missing value, we set the missing value to the mean of these two values for continuous variables. For non-continuous variables, we randomly chose a trait value from the year before or year after the missing year. We adopted this approach based on the assumption that aging is typically a gradual process, and hence, an individual’s traits in 1 year are likely predictive of trait values in the previous or next year. This backward- and forward-filling approach provided values for 10% of female-trait-years.

Second, for all other missing data (16% of female-trait-years), we used predictive mean matching to females’ annual values using the *aregImpute* function from the Hmisc package [59]. Importantly, creating the age-predicting clock itself (see below) required us to split our data into “training” and “test” sets to evaluate generalization error. To avoid leaking information between these data sets, we split the data set into training and test sets before performing predictive mean matching. Specifically, we created five training data sets that contained 80% of the data and five corresponding test data sets that each contained the remaining 20% of the data. Observations from each individual were evenly distributed across the five test data sets, such that a given individual might appear in its own training set. We then used the *aregImpute* function to impute the missing data in each training and test data set separately. Predictive mean matching uses a weighted probability draw to select among a set of predicted values calculated for the missing data point’s “closest neighbors” (i.e., other rows in the data frame that have similar values for other predictor variables [59]). Female age is not included in this data frame and is not considered part of the predictive mean matching process. This process was repeated for each piece of missing data. We repeated the imputation process five times for each piece of missing data in each training and test set, producing five imputations per set (i.e., 25 total pairs of training-test sets, representing five training-test splits and five imputation procedures per split).

### Building the NPB age-predicting clock

We used the *train* function within the caret package to build the NPB age-predicting clock using random forest regression (see Supplementary Information for comparisons against two alternatives, elastic net regression and Gaussian process regression; [60]). We set the number of trees (ntree) to 2000 and defined the optimal number of variables to randomly sample as candidates at each tree (mtry) as the value that maximized *R*^2^ between predicted and known age across all samples in the training set. The predictor variables were the 49 centered and standardized age-associated traits measured annually for each female, and the response variable was each female’s known chronological age. The five imputation sets (described above) were used to predict each female’s age five times, which were averaged to produce her final age prediction. We assessed performance by evaluating how much variance in predicted age was explained by known chronological age (via *R*^2^) and the median error in the predicted age estimates for all female-years. To understand if females were consistently predicted to be younger or older than their known ages, we calculated the repeatability of “relative NPB age” as the residuals of a linear model regressing predicted against known age. Relative NPB age represents how much older or younger an individual was predicted to be, on average, compared to her true age, with positive values indicating females who were predicted to be older for their known age and negative values indicating females who were predicted to be younger than their known age.

In addition to the main NPB age-predicting clock, we also created a variant clock (“NPB-restricted”) that excluded 17 traits already known to explain variation in female mortality in the Amboseli baboons: fecal glucocorticoid concentrations (one of the endocrine traits in category 2), percent of time being groomed (one of the activity budget traits in category 1), and all traits under social integration and dyadic sociality (all traits in categories 7 and 8; Table S1; [44, 61, 62]). The NPB-restricted clock also excluded the incidence of puncture wounds (one of the illness and injury traits in category 3) because the loss of the other variables prevented us from imputing this trait (this trait also had low importance in the main NPB clock). NPB-restricted was calibrated using the same steps described above.

### Statistical analyses on NPB clock predictions

Clocks that measure biological age should ideally predict aging-related outcomes, such as all-cause mortality, beyond the accuracy possible from chronological age alone. To test this possibility, we created a Cox proportional hazards model using the survival package in R [63]. The predictor variable for this model was lifetime relative NPB age, calculated as the average of all of a female’s relative NPB age estimates across adulthood (relative NPB ages are the residuals of a linear model regressing predicted against known age). The data set included 319 females with 162 deaths and 157 right-censored individuals who were still alive at the time of analysis or who were dropped from regular observations prior to death.

Because fluctuations in biological age across adulthood might also predict mortality risk within a given year, we also created a time-varying Cox proportional hazards model to test if females who looked old-for-age in a given year of life experienced higher mortality risk in that year. The predictor variable in this model was each female’s annual measure of relative NPB age. Female identity was included as a clustering variable. The data set for this analysis included 2402 female-years from 319 females. The number of known deaths in this analysis was 138, lower than for the model using lifetime relative NPB age described above, because 24 females had < 60 days of data in their last year of life, so they were filtered out of the data set (see above).

We repeated our Cox proportional hazards models using age predictions from the NPB-restricted clock, which excluded traits known to predict mortality (discussed above). We ran both a “lifetime” version of the model which used each female’s average relative NPB-restricted age as the sole predictor variable and a time-varying version that included each female’s annual measure of relative NPB-restricted age. As above, female identity was included as a clustering variable. Sample sizes were the same as the two parallel analyses for the main NPB clock, described above.

We next tested our expectation that early-life adversity increases relative NPB age. To do so, we created two linear mixed models using the lme4 package in R [57]. In both models, the response variable was each female’s delta age (Age_Δ_) in a given year of life, defined as the predicted age from the NPB clock minus known chronological age in years. Because this variable is correlated with known age, we also included known chronological age as a fixed effect in the model. The two models differed in whether each of the six sources of adversity was modeled (1) independently in a multivariable framework or (2) cumulatively using an index that summed the six sources of adversity a female could experience in the first four years of life. For the multivariable version, group size, maternal rank, and maternal social connectedness were modeled as continuous variables, while maternal death, close-in-age sibling, and drought were modeled as binary variables. For the cumulative index, following [45], continuous variables were converted to binary variables and scored as present if the female’s experience fell in the most adverse quartile of the population distribution (see Supplementary Information). Both models also included three variables that captured the female’s current social and environmental conditions in a given year of life: (i) average proportional dominance rank in that year, (ii) average number of adult females in her social group in that year as a measure of social density and resource competition, and (iii) yearly rainfall anomaly, which represents the relative amount of rainfall in that year compared to all other years throughout the history of the ABRP project (see Supplementary Information). Individual identity was modeled as a random effect in both models. The sample sizes for these early adversity models were smaller because we were missing information on early-life experiences for some females (*N* = 256 females with 108 known deaths for the lifetime model; *N* = 256 females over 1867 total years with 92 known deaths for the annual model).

Finally, we tested whether relative NPB age predicted female mortality, controlling for exposure to early-life adversity. The lifetime and time-varying versions of these models were run using the same procedures described above. Our analysis was conducted on a subset of the full data set (*N* = 256 females with 1867 female-years of observation).

## Results

### Many physiological and behavioral traits change with female age

**Fig. 1:**
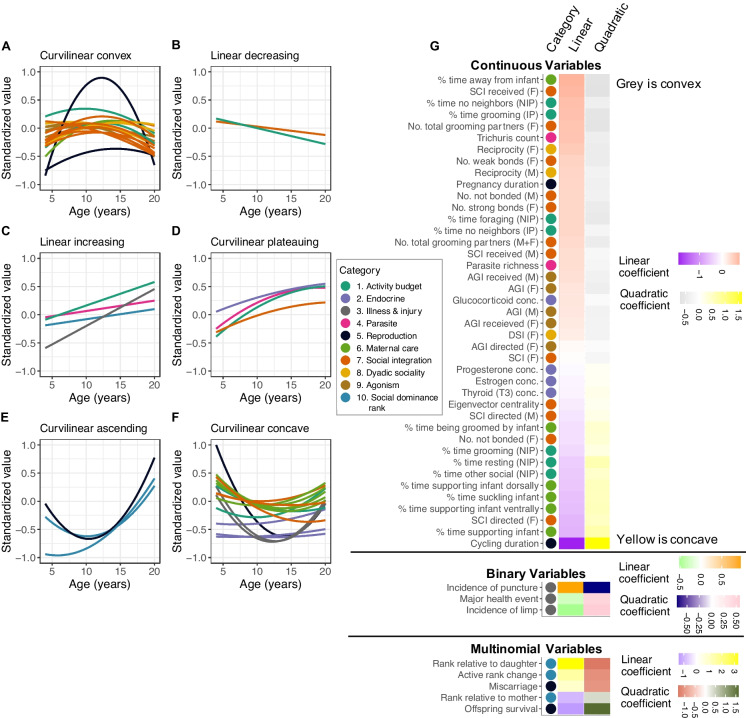
Forty-nine traits exhibited significant linear or curvilinear relationships with female age. **A**–**F** Standardized values of each of the 49 traits that had statistically significant relationships with age are shown on the *y*-axis, as a function of female age in years on the *x*-axis (coefficients for linear and quadratic relationships with female age for each trait are in Table S3; see Figs. S3 and S4 for visualizations of relationships for each trait separately). Traits are grouped by the nature of their relationship with age: **A** curvilinear convex, **B** linear decreasing, **C** linear increasing, **D** curvilinear plateauing, **E** curvilinear ascending, and **F** curvilinear concave. Colors indicate the type of trait being modeled (Table S1). For continuous variables, we plotted predicted fits based on the linear and quadratic age terms from the best-fitting linear models. For categorical variables, the figure shows quadratic fits to the raw data for visualization purposes only; our actual statistical models were based on binomial or multinomial generalized linear models (Table S3). **G** A series of heatmaps of the linear and quadratic coefficients for the 49 traits grouped into continuous traits with Gaussian error distributions and categorical traits modeled using binomial or multinomial models. Colored dots correspond to the category of the trait being modeled (Table S1). Linear coefficients represent the predicted per-year change in the trait value in standard deviations (i.e., the slope). Quadratic coefficients represent the predicted change in slope over time, with positive coefficients representing concave or ascending slopes and negative coefficients representing convex or plateauing slopes. For continuous traits, the coefficients are extracted directly from the linear model. For the categorical traits, coefficients represent the log odds of each variable

We found that 63% (49 of 78) of female traits exhibited significant linear or quadratic relationships with female age (Fig. 1; Table S3). The traits most strongly associated with age were for measures of social dominance rank, reproduction, maternal care, and social integration (Fig. S3; Fig. S4; Table S3). In terms of social dominance rank, older females were more likely to outrank their mother, be outranked by an adult daughter, and exhibit an “active” fall in rank (i.e., a change in rank not solely due to demographic events like a change in the number of females in the hierarchy; Fig. S3; Table S3). In terms of reproduction and maternal care, young and old females experienced longer periods of ovarian cycling before conceiving than middle-aged females (Fig. S3; Table S3). Young and old mothers also had the lowest offspring survival, and in the analysis of pregnancy outcomes, older females were more likely to experience fetal loss or stillbirth than younger females (Fig. S3; Table S3). Young and old mothers spent the most time physically supporting and nursing their infants (Fig. S3; Table S3). In terms of sociality, old females were less likely to initiate and receive grooming with other females. They also had the fewest distinct grooming partners, had the lowest eigenvector centrality within their group’s grooming network, spent less time grooming, and spent more time without a neighbor within 3 m (Fig. S3; Table S3).

Several traits were not associated with age. Age did not explain several components of female activity budgets, including the percentage of time females spent feeding, walking, or standing (Table S3). Age was also not associated with the strength of female relationships with adult males, including the aggregate strength of grooming relationships with all adult males in the group (SCI-M) and the strength of dyadic grooming relationships with males (DSI-M). Female age also did not explain the incidence of observable signs of illness (with the exception of parasite richness and whipworm burdens).

### The NPB clock predicts individual age in wild female baboons

**Fig. 2:**
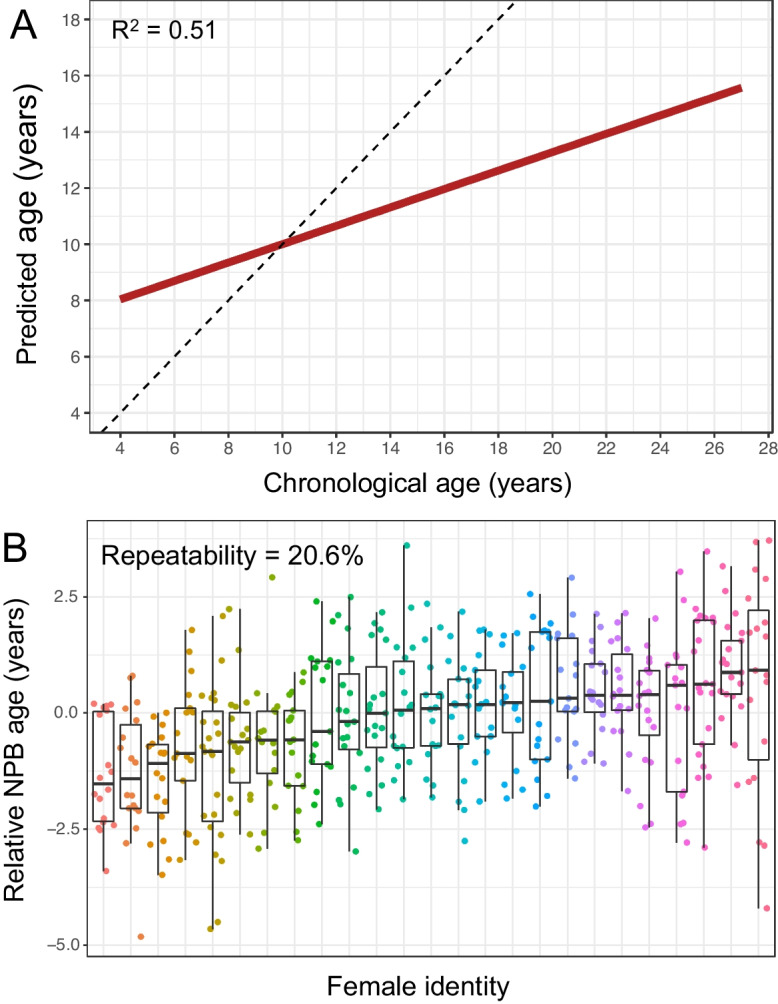
The NPB age-predicting clock in wild female baboons. **A** Predicted ages from the random forest NPB clock, plotted against known chronological age. The dashed line represents the 1:1 relationship between predicted and chronological age; the red line shows the fit of a linear model relating these two variables. Age predictions were compressed relative to the 1:1 line. **B** Relative NPB age for the 25 female baboons who had the most years of data in our data set (16–23 age predictions per female; see Fig. S1 for numbers of years of data per female). Relative NPB age is calculated as the residuals of a linear model regressing predicted against the female’s chronological age. The repeatability of relative NPB age was 20.6%

Our NPB clock explained 51% of the variance in females’ known ages (Fig. 2A) and had moderate repeatability of relative NPB age (20.6%; Fig. 2B; Table S5). The age predictions were compressed relative to the 1:1 line, systematically over-predicting the ages of young individuals and under-predicting the ages of old individuals (Fig. 2; Fig. S5). The five traits with the highest importance to the NPB clock were a female’s social rank relative to her daughters and her mother, the duration of ovarian cycling she exhibited between pregnancies, the percentage of time she spent with no neighbors, and her social connectedness to other adult females (Fig. S6). Compared to other age predictors in Amboseli, the NPB clock produced more accurate age estimates than a microbiome clock, early and late-aged body mass index (BMI), differential white blood cell counts from blood smears, and blood cell composition by flow cytometry (Fig. S7; [20]). However, the NPB clock was less accurate than a DNA methylation-based epigenetic clock (*R*^2^ = 0.60 in females, median error = 1.62 years) and dentine exposure (*R*^2^ = 0.85, median error = 1.62 years; [20]).

### Older biological age in the NPB clock predicts adult female survival

**Fig. 3:**
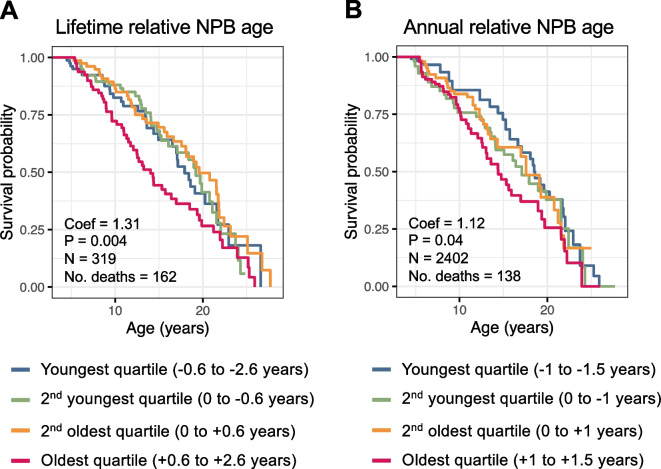
Relative NPB age predicts adult female survival. Plots show female survival as a function of **A** mean lifetime relative NPB age or **B** relative NPB age in each year of life. Colors represent quartiles of lifetime and annual NPB age estimates. Blue represents females who look the youngest for their age, and pink represents females who look oldest for their age

Females reliably predicted to be older than their true chronological age (Fig. 2B) might also exhibit higher mortality. In support of this possibility, lifetime relative NPB age predicted adult female survival (Fig. 3A; hazard ratio = 1.31; 95% CI = 1.09–1.58; *P* = 0.004; *N* = 319). This effect was driven by females in the oldest quartile of NPB age predictions, whose relative NPB ages were, on average, > 0.6 years older than their known ages. These females led lives that were typically 4 years shorter than females in the other quartiles of lifetime relative NPB age (Fig. 3A, pink lines), with median lifespans of 14.0 years (95% CI = 12.1–18.5). Our results also support the idea that fluctuations in biological age across adulthood predict mortality risk. Indeed, our time-varying Cox proportional hazards model revealed that, for every year older a female’s relative NPB age was compared to her known age, her risk of death in that year increased 12% relative to baseline for her age class (Fig. 3B; hazard ratio = 1.12; 95% CI = 1.01–1.24; *P* = 0.04, *N* = 2402; see [64] for age-specific hazards for females; median annual adult mortality for female baboons in Amboseli = 0.093; range = 0.034–1.00 [64]).

These patterns were not driven by the inclusion of mortality-associated traits in the NPB clock. The NPB-restricted clock was similarly successful in predicting female age as compared to the main NPB model (*R*^2^ = 0.49 vs *R*^2^ = 0.51; Fig. S8). Pearson’s correlation between age predictions from the main NPB clock and the NPB-restricted clock was 0.93. Further, lifetime relative age calculated from the NPB-restricted clock predicted female mortality with a similar effect size to relative age estimates from the main NPB clock (Fig. S9A vs Fig. 3A; lifetime relative age from NPB-restricted clock: hazard ratio = 1.29; 95% CI = 1.09–1.52; *P* = 0.003; lifetime relative age from main NPB clock: hazard ratio = 1.31; 95% CI = 1.09–1.58; *P* = 0.004). Similar to the main NPB clock, annual relative age estimates from NPB-restricted clock also predicted survival (hazard ratio = 1.10; 95% CI = 1.01–1.19; *N* = 2402; *P* = 0.04; Fig. S9B).

### Early-life adversity is linked to old-for-age NPB clock predictions

**Fig. 4:**
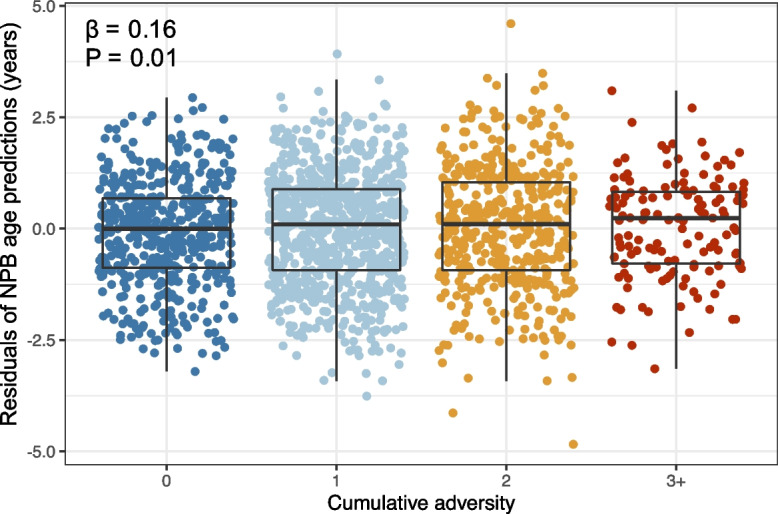
Early-life adversity is linked to an increase in NPB age predictions. This plot shows the residuals of NPB predicted age (*y*-axis) as a function of cumulative early-life adversity (*x*-axis), in a model controlling for chronological age, proportional rank, rainfall anomaly, and group size. Individuals who experience three or more sources of adversity in early life appear, on average, 0.48 years older for age in any year of life compared to individuals who do not experience any sources of adversity in early life (Table S6; *β* = 0.16, *P* = 0.01, *N* = 1867). However, this relationship is noisy: many individuals match their age predictions, and some appear young-for-age (*y*-axis values < 0); similarly, some females who experienced little early-life adversity also appear old-for-age

Harsh environmental conditions in adulthood were linked to old-for-age NPB clock estimates. Females who experienced more adverse conditions in early life had NPB clock age predictions that were slightly older-for-known age than females who experienced fewer sources of early-life adversity (Fig. 4; Table S6; *β* = 0.16; *P* = 0.01; *N* = 1867 female-years). Females who experienced three or more sources of adversity were predicted to be, on average, 0.48 years older-for-age in a given year of adulthood compared to individuals who experienced no sources of early-life adversity. However, the relationship between early-life experiences and NPB clock predictions was noisy, and no individual source of early-life adversity significantly predicted NPB clock estimates (Fig. 4; Fig. S10; Table S7). Further, NPB clock age estimates were also not predicted by current social or environmental conditions, including the female’s proportional dominance rank, living in a large social group, or a year with low rainfall (Tables S6 and S7).

Relative NPB age did not predict female mortality after accounting for early-life experiences. Consistent with prior analyses [45, 47, 48], early-life adversity was a strong predictor of female longevity (Table S8; HR = 1.40; 95% CI = 1.11–1.75; *P* = 0.004). However, including early-life adversity as a covariate reduced the predictive power of relative NPB age on female mortality, perhaps because relative NPB age is modestly correlated with early-life adversity. The hazard ratio for lifetime relative NPB age dropped from 1.31 (95% CI = 1.09–1.58) to 1.21 (95% CI = 0.97–1.50), and the confidence interval for the hazard ratio overlapped one (*P* = 0.10; Table S8). We found a similar attenuation of effect in a time-varying model of annual values of relative NPB age (Table S9).

## Discussion

Observational data from wild primates may reflect individual differences in biological age. The NPB clock we created produced biologically relevant age predictions for female baboons: females predicted to be older than their known ages across adulthood tended to lead shorter lives than individuals whose age predictions were, on average, younger than their true ages. Age predictions in a single year of life also predicted mortality risk in that year, indicating that annual variation in biological age could contribute to fluctuations in mortality risk across adulthood. Our NPB clock even predicted mortality when we removed features with previously known links to female longevity in our population [44, 61, 62], even though some of these features had high feature importance in the clock. Hence, biological age in female baboons is reflected in a variety of behavioral and physiological markers. Notably, the NPB clock did not predict mortality after controlling for early-life experiences. This result suggests that the effects of early adversity on biological age may partly explain the connection between NPB clock age predictions and female mortality.

Our clock expands the methodological approaches for measuring biological age in long-term studies of natural animal populations. Compared to the diversity of age-predicting clocks in the literature, our clock shares the most in common with “frailty clocks” developed for humans and laboratory mice that use measures of individual ability to perform activities of daily living [26, 36, 65]. Frailty indices are among the most powerful predictors of all-cause mortality in humans, and our work helps extend these findings to non-human animals [35–38], including populations for which fine-grained data are available on the social and environmental conditions individuals experience across life (e.g., [20, 21]). In the current study, we found that early-life adversity—one of the strongest environmental predictors of lifespan and mortality risk in this population [45, 47, 48]—was linked to old-for-age predictions in the NPB clock. Female baboons who experienced three or more sources of adversity in the first four years of life were predicted to be about 6 months (0.48 years) older than their true age in any year of adulthood compared to females who experienced no known sources of early-life adversity. This effect size would translate to a loss of 2–3 years of adult lifespan, given the relationship between NPB age and mortality we observed.

Our results join several previous studies in humans, using both frailty indices and epigenetic clocks, which find that individuals who experienced early-life adversity appear old-for-age in adulthood (e.g., [66–71]). Our results differ somewhat from two other age-predicting clocks created for the Amboseli baboon population. For instance, for female baboons, epigenetic age in a given year of adulthood was not predicted by either cumulative early-life adversity or current social dominance rank [20]. An unpublished age-predicting clock based on gut microbiome composition found weak and inconsistent effects of early-life adversity on microbiome age, but in adulthood, females had old-for-age microbiome compositions in the dry season—a period linked to resource deprivation. These two clocks and the NPB clock together suggest that different biological systems respond differently to socio-environmental exposures and reflect different aspects of biological aging. For instance, non-invasive clocks that include several behavioral phenotypes, like the NPB clock, might be best for tracing longitudinal changes in biological age within individuals as they experience a variety of socio-environmental challenges across life. Such clocks are probably best for measuring aspects of aging linked to tasks of daily living and changing social relationships. By contrast, given the increased difficulty of collecting typically used sample types, epigenetic clocks might be better suited to cross-sectional questions that address mechanistic aspects of biological age within specific tissues and organs (e.g., blood, muscle, brain). These ideas are consistent with prior research in humans that suggests that different types of clocks—even those based on the same data type, but optimized to predict different outcomes (e.g., chronological age versus impending mortality versus the pace of aging)—are linked to different aspects of aging (e.g., [28, 37, 38, 72, 73]).

Interestingly, the most important feature in our age predictions was females’ social rank relative to their daughters: as age increases, the probability that a female ranks below one or more of her daughter(s) increased exponentially. Similarly, the third most important trait in the NPB clock is females’ social rank relative to their mothers: as age increases, the probability that a female will rank above her mother increases linearly. Baboon females in Amboseli follow nepotistic rank ordering, such that female offspring generally rank immediately below their mothers [74, 75]. However, they may eventually come to rank above their mother: in 34% of mother-daughter pairs in Amboseli, the daughter ranked above her mother at some point in adulthood, and this switch was more likely to be observed for older mothers [75]. If mother-daughter rank changes occur because age-related physical declines lead mothers to either lose their competitive ability [76] or cede rank to their daughters in “consensual” rank reversals [77, 78], then these explanations may also explain the association between rank reversals and the NPB clock.

The duration of ovarian cycling before conceiving was also important to the NPB clock’s age predictions (Fig. S6). In Amboseli, females cycle a median of nine times between menarche and their first pregnancy [79]. After their first pregnancy, they experience fewer ovarian cycles between pregnancies, on average, and this number is relatively stable throughout prime adulthood (median of approximately four cycles; [80]). However, once they reach late adulthood (around 18 years of age), the number of cycles between pregnancies increases again as females undergo reproductive senescence [81].

While our clock illustrates the use of observational data to measure biological age in wild animals, it relies on many data sets that are either unique to the Amboseli baboon population or are collected using different methods across populations. Consequently, it cannot be directly extrapolated to other non-human primate or mammal populations, and we cannot easily test the generalizability of this specific clock across species and populations. Unfortunately, the lack of uniform methods across long-term studies of wild mammals likely makes a more universally applicable NPB clock impossible. Despite this limitation, the NPB clock serves as an example of how long-term, non-invasive data can be used in research on comparative aging.

In conclusion, physiological and behavioral traits measured across the life course can be used to predict age in a wild long-lived primate. This metric of biological age predicts lifespan and is, itself, predicted by the number of cumulative hardships that individuals experience in early life. Indices of biological age created with physiological and behavioral traits share some similarities with visual assessments of aging and frailty indices, and a diversity of age-predicting clocks is likely to be important for measuring aging in different biological systems. Age-predicting clocks like ours offer opportunities to study aging non-invasively and will be most useful for long-term animal studies that already have longitudinal data across individuals’ lifespans.

### Supplementary Information

Below is the link to the electronic supplementary material.

Supplementary file1 (XLSX 55 KB)

Supplementary file2 (DOCX 5248 KB)

## Data Availability

All data and code are available at https://github.com/cweibel2/npb_age_clock.
