# Correction to: Using non‑invasive behavioral and physiological data to measure biological age in wild baboons

**DOI:** 10.1007/s11357-024-01249-2

**Published:** 2024-06-20

**Authors:** Chelsea J. Weibel, Mauna R. Dasari, David A. Jansen, Laurence R. Gesquiere, Raphael S. Mututua, J. Kinyua Warutere, Long’ida I. Siodi, Susan C. Alberts, Jenny Tung, Elizabeth A. Archie

**Affiliations:** 1 Department of Biological Sciences, University of Notre Dame, Notre Dame, IN USA; 2 Department of Biology, Duke University, Durham, NC USA; 3 Amboseli Baboon Research Project, Amboseli National Park, Kajiado, Kenya; 4 Department of Evolutionary Anthropology, Duke University, Durham, NC USA; 5 Duke University Population Research Institute, Duke University, Durham, NC USA; 6 Department of Primate Behavior and Evolution, Max Planck Institute for Evolutionary Anthropology, 04103 Leipzig, Germany; 7 Canadian Institute for Advanced Research, Toronto, M5G 1M1 Canada; 8 Faculty of Life Sciences, Institute of Biology, Leipzig University, Leipzig, Germany

**Correction to: GeroScience**

10.1007/s11357-024-01157-5

The original version of this article unfortunately contained an error in Figure 2.

Figure 2a captured incorrectly from this article; the figure should have appeared as shown below.

Incorrect Figure 2


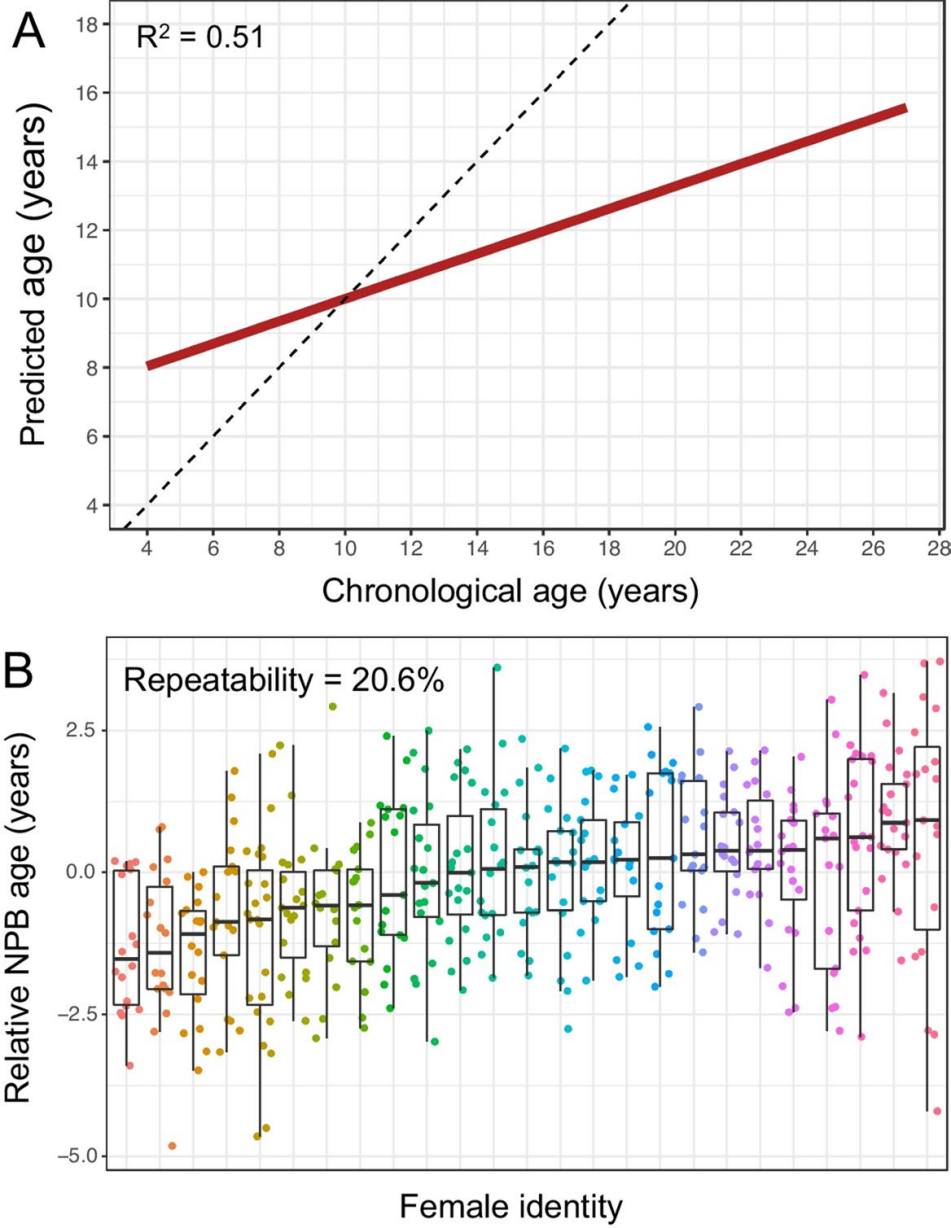


Correct Figure 2

**Fig. 2:**
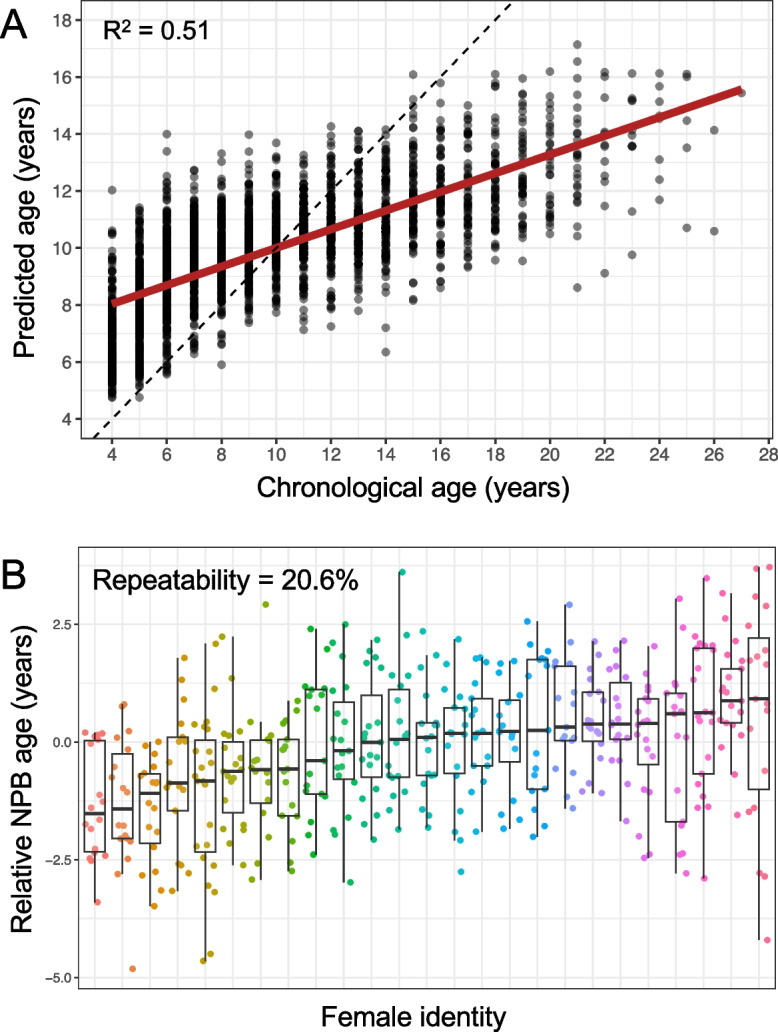
The NPB age-predicting clock in wild female baboons. A Predicted ages from the random forest NPB clock, plotted against known chronological age. The dashed line represents the 1:1 relationship between predicted and chronological age; the red line shows the fit of a linear model relating these two variables. Age predictions were compressed relative to the 1:1 line. B Relative NPB age for the 25 female baboons who had the most years of data in our data set (16–23 age predictions per female; see Fig. S1 for numbers of years of data per female). Relative NPB age is calculated as the residuals of a linear model regressing predicted against the female’s chronological age. The repeatability of relative NPB age was 20.6%

