# Supplementary material for: Using non-invasive behavioral and physiological data to measure biological age in wild baboons
Source: GeroScience. 2024 May 2;46(5):4059–74. doi: 10.1007/s11357-024-01157-5 (PMC11336142; doi:10.1007/s11357-024-01157-5)
Supplement: Supplementary file 2 — Supplementary file2 (DOCX 5248 KB) [file 11357_2024_1157_MOESM2_ESM.docx]

**Supplementary information for “Using non-invasive behavioral and physiological data to measure biological age in wild baboons”**

Chelsea J. Weibel^1^, Mauna Dasari^1^, David A. Jansen^1^, Laurence R. Gesquiere^2^, Raphael S. Mututua^3^, J. Kinyua Warutere^3^, Long’ida I. Siodi^3^, Susan C. Alberts^2,4,5^, Jenny Tung^2,4.5,6,7,8^, Elizabeth A. Archie^1^

^1^ Department of Biological Sciences, University of Notre Dame, Notre Dame, IN, USA

^2^ Department of Biology, Duke University, Durham, NC, USA

^3^Amboseli Baboon Research Project, Amboseli National Park, Kenya

^4^ Department of Evolutionary Anthropology, Duke University, Durham, NC, USA

^5^ Duke University Population Research Institute, Duke University, Durham, NC, USA

^6^ Department of Primate Behavior and Evolution, Max Planck Institute for Evolutionary Anthropology, 04103 Leipzig, Germany

^7^ Canadian Institute for Advanced Research, Toronto, Canada M5G 1M1, Canada

^8^ Faculty of Life Sciences, Institute of Biology, Leipzig University, Leipzig, Germany

^*^Correspondence to [earchie@nd.edu](mailto:earchie@nd.edu)

**Table of contents**

**Methods**

1. Measuring age-related traits in female baboons
2. Measuring covariates of age-related traits
3. Measuring early-life adversity
4. Comparing regression approaches for building the NPB clock

**Figures**

Fig. S1 – Annual physio-behavioral data for female baboons

Fig. S2 – Clock performance with less missing data

Fig. S3 – Age patterns for 49 traits – ordered by appearance in **Fig. 1B** and **Table S3**

Fig. S4 - Age patterns for 49 traits – ordered by the shape of their relationship with age in **Fig. 1A-1F**

Fig. S5 – Comparison of random forest, elastic net, Gaussian process clocks

Fig. S6 – Feature importance in the NPB clock

Fig. S7 – Comparing the NPB clock to other ABRP age-predicting clocks

Fig. S8 – Age predictions for NPB-restricted

Fig. S9 – Survival plots for NPB-restricted

Fig. S10 – Individual sources of adversity and NPB age predictions

**See Supplementary Tables excel document**

Tables S1-S9

**References**

**Supplementary Methods**

1. Measuring age-related traits in female baboons

The methods below describe how we measured the 78 female traits described in Table S1 and that were tested for linear and quadratic relationships with age.

***Category 1: Female activity budgets (blue-green traits in Table S1)*.** During regular monitoring visits, observers conduct 10-minute focal follows of adult females, during which they perform point sampling at 1-minute intervals where they record information on the subject’s activity (feeding, resting, walking, grooming), posture (standing or sitting), and whether the subject has adult neighbors within 5 meters (Alberts et al., 2020). Using these data, we generated 18 variables that capture the percent of time focal females spent engaging in several activities (blue-green rows in **Table S1**). We calculated activity budgets separately depending on whether females had a dependent infant < 70 weeks of age (IP = infant present) or did not have a dependent infant (NIP = no infant present) because the presence of an infant can profoundly affect female activities and neighbors. To calculate activity budget data for a given female in a year of life, we filtered to years when the female had at least 100 point samples. Because females might spend some time with and without a dependent infant in a given year of life, some females have both IP and NIP activity budgets in a given year. The activity budgets we collected both in the presence (IP) and absence (NIP) of infants include: *percentage (%) time being groomed*, *% time with no neighbors* (other than the subject’s infant, within 5 meters), *% time feeding*, *% time foraging* (includes feeding and searching for or processing food), *% time grooming*, *% time engaging in other social behaviors*, *% time resting*, *% time standing*, and *% time walking.*

***Category 2: Endocrine (purple traits in Table S1)*.** Endocrine traits were the concentrations of three steroid hormone and one thyroid hormone in non-invasively collected fecal samples. Fecal samples were collected ad libitum starting in 2000. Fecal sample collection, storage, and extraction were performed as described previously (Khan et al., 2002; Lynch et al., 2003). The steroid metabolites we measured were *estrogen (fE), progesterone (fP), glucocorticoid (fGC),* and the thyroid hormone was *triiodothyronine (mT3)*; all were measured via radioimmunoassay (Gesquiere et al., 2005, 2008, 2018). In total, the dataset contained 13,399 fE samples, 11,004 fP samples, 13,913 fGC samples, and 6,764 mT3 samples.

***Category 3: Illness and injury (grey traits in Table S1)*.** During daily monitoring visits, observers record observable wounds, signs of illness, and locomotor impairment. From these data, we constructed seven binary variables that describe whether an individual experienced an illness or injury in a given year of life. Several of these variables capture incidences of specific injuries (*incidence of limp, incidence of puncture wound, incidence of a linear cut or slash,* and *incidence of scape)*, while *incidence of injury* describes whether the individual experienced any type of physical injury in a given year. *Incidence of illness* describes whether the individual exhibited any symptoms of disease or pathologies in a given year (e.g., diarrhea, vomiting, fatigue, weakness, coughing, sneezing, etc.). Finally*, incidence of major health event* describes whether the individual experienced an illness or injury in a given year of life that impaired their locomotion.

***Category 4: Parasite (pink traits in Table S1)****.* We measured two aspects of individual parasite loads from fecal samples via standard fecal flotation and sedimentation techniques. *Trichuris count* is a count of *Trichuris trichiura* (whipworm) eggs in 4 grams of fecal sample, and *parasite richness* is the number of distinct parasite taxa observed in a fecal sample, including *T. trichiura,* strongyles, *Abbreviata caucasica, Streptopharagus pigmentatus, Strongyloides fulleborni, Enterobius vermicularis, Primasubulura sp., Acanthocephalan sp.,* and an unknown trematode species. Parasite counts were performed on fecal samples starting in 2011. Each sample is homogenized and stored in 10% buffered formalin and then gastrointestinal helminth egg counts were performed using standard sugar flotation and sedimentation protocols (see (Akinyi et al., 2019; Habig et al., 2019) for details).

***Category 5: Reproduction (black traits in Table S1)*.** We recorded three types of reproductive traits: duration of reproductive states (including cycling, pregnant, and postpartum amenorrhea), pregnancy outcome, and offspring survival.

*Duration of reproductive states.* Females were assigned to one of three reproductive states—ovarian cycling, pregnancy, or postpartum amenorrhea—based on the characteristics of their perineal sexual skins, the color of their paracallosal skins, and the presence of external menstrual bleeding, which were recorded during near-daily observational visits (Altmann, 1973; Gesquiere et al., 2007; Shaikh et al., 1982; Wildt et al., 1977). Durations were calculated as the number of days between the following time points: for pregnancy, from conception to live birth; for postpartum amenorrhea, from live birth to the resumption of cycling; and for cycling, from the attainment of menarche or resumption of cycling after a previous pregnancy to conception. Conception is identified by the cessation of ovarian cycling and a change in the color of the paracallosal skin from grey/black to pink (Altmann, 1973). We only considered the duration of pregnancies that resulted in the birth of a live offspring, because miscarriages almost always result in shorter pregnancy durations. Similarly, we only considered the duration of postpartum amenorrhea when the offspring survived at least 70 weeks. Seventy weeks represents the approximate age at weaning in our population, and if offspring do not live to weaning, baboon mothers will rapidly return to ovarian cycling (Altmann, 1998).

*Pregnancy outcome.* For each female’s year of adult life (from birthdate to birthdate), we identified the outcome of each conception. This variable is a three-level factor, with the levels representing: 1) whether she conceived and gave birth to a live offspring, 2) whether she conceived and miscarried, or 3) whether she did not conceive at all in that year. The variable for each year of life corresponds with the date of conception (e.g., if an individual conceives at 6.7 years and gives birth at 7.2 years, the pregnancy outcome will be counted for her 6^th^ year).

*Offspring survival.* For each female’s year of adult life (from birthdate to birthdate), we identified the outcome of each live birth. This variable is a three-level factor, with the levels representing: 1) an offspring she gave birth to survived to weaning (70 weeks), 2) whether the offspring to whom she gave birth died before reaching 70 weeks, or 3) whether she did not give birth to a live offspring in that year. The variable for each year of life will correspond with the date of the offspring’s birth (e.g., if an individual gives birth at 7.2 years, the outcome of that birth will be counted for her 7^th^ year).

***Category 6: Maternal care (light green traits in Table S1)*.** These traits were collected using the same methods described under the activity budget traits above. Briefly, measures of maternal care variables were collected during 10-minute focal follows. In addition to recording the subject’s activity, position, and proximity to other individuals (see above), observers also recorded information on the individual’s interactions with their infants during 1-minute point samples. These data were used to generate seven variables that represent maternal care, including: *percentage (%). time away from infant* (not in physical contact with), *% time grooming infant, % time being groomed by infant*, *% time infant suckling*, *% time supporting infant dorsally*, *% time supporting infant ventrally*, and *% time supporting infant* (dorsally or ventrally combined). We quantified these measures during periods of time when subjects had a dependent juvenile that was less than 70 weeks old (the approximate age of weaning; Altmann, 1998) and when we had 100 or more point samples available for the individual during this time period.

***Category 7: Social integration (orange traits in Table S1)*.** Sociality measures are determined based on grooming interactions between focal individuals and all other individuals in their social group in a given year of life collected during representative interaction sampling. During representative interaction sampling, observers move through a baboon group recording all visible observations of grooming, while conducting 10-min focal animal follows on a randomized, preselected list of females (Alberts et al., 2020; Archie et al., 2014). We created 18 different metrics of overall sociality which capture the number and type of bonds focal females held, composite sociality indices that captured overall grooming rates (see below), and a measure of the subject’s centrality in her group’s social network. Variables containing an (F) represent bonds with adult females, variables with an (M) represent bonds with adult males, and variables with (M+F) represent bonds with both adult males and females.

We created 11 variables that capture the number and strength of a female’s bonds with other individuals in her social group in a given year of life; they include; they include: *number (no.) not bonded (F), no. not bonded (M), no. weak bonds (F), no. weak bonds (M), no. strong bonds (F), no. strong bonds (M), no. very strong bonds (F), no. very strong bonds (M), no. total grooming partners (F), no. total grooming partners (M), no. total grooming partners (M+F)*. To determine the number of individuals that fell into each category, we determined the relative strength of the grooming relationships for every dyad in the group throughout that female-year of life, controlling for observer effort. The number of individuals not bonded represents the number of individuals that the individual did not groom with at all, the number of individuals with a weak grooming interaction represent those in the bottom 50% of grooming interactions for the whole group, the number of individuals with a strong grooming interaction represent those in the top 50% of grooming interactions for the whole group, and the number of individuals with a very strong grooming interaction represent those in the top 10% of grooming interactions for the whole group.

We created 6 composite social connectedness indices (SCI). Our measurements of social connectedness are based on a standardized index that measures the relative frequency with which the individual groomed and/or was groomed by other individuals in the social group, compared to the grooming rates of all other females alive in the population in the same female-year of life, adjusted for observer effort (Archie et al., 2014). *SCI (M)* and *SCI (F)* are composite measures that take into consideration all grooming interactions in which the individual participated—including giving and receiving grooming; *SCI directed (F)* and *SCI directed (M)* are composite measures that only take into consideration interactions in which the individual groomed its partner; *SCI received (F)* and *SCI received (M)* are composite measures that only take into consideration grooming interactions in which the individual was groomed by others.

*Eigenvector centrality*. Eigenvector centrality is a variable that captures the centrality of the subject to the social network of the group. This variable takes into consideration all of the subject’s grooming interactions, as well as the interactions of all other members of the group and measures the extent to which an individual’s partners are connected to others in the network. Each individual’s measure of centrality is the sum of the centrality values of the nodes to which it is connected. Those with high eigenvector centrality have connections with partners who themselves have a large number of partners (Cheney et al., 2016). We calculated eigenvector centrality in each month and then averaged it across each individual’s years of life.

***Category 8: Dyadic sociality (yellow traits in Table S1)*.** Dyadic sociality variables are constructed using data on grooming interactions collected during representative interaction sampling (described above). These variables are similar to the composite sociality indices (SCI; (Archie et al., 2014)), but rather than capturing overall social relationships, these metrics capture the relative strength and reciprocity of individuals’ grooming relationships with their top three social partners. Variables containing an (F) represent bonds with adult females and variables with an (M) represent bonds with adult males.

*DSI (F)* and *DSI (M)*. DSI stands for dyadic sociality index and these measures capture the bond strength of each female’s top three social bonds (with females and males, respectively), relative to all other females alive in the population at the same time, adjusted for observer effort.

*Reciprocity (F)* and *Reciprocity (M)*. Reciprocity is a measure of the interaction asymmetry for the individuals top three social partners. Interaction asymmetry occurs when one of the partners in the dyad spends more time grooming their partner than vice versa.

***Category 9: Agonism (brown traits in Table S1)****.* Data on females’ agonistic interactions are collected via representative interaction sampling during all monitoring visits (described above; (Alberts et al., 2020). The agonistic interactions we considered here include non-contact displays of aggression (e.g., raised brow, open mouth display, threat yawn), physical contact (e.g., hit, push, bit), displacements, and cases where the recipient submitted without an observed aggressive act. Our agonistic variables are based on a standardized index that measures the relative frequency with which the individual received and/or initiated aggressive interactions with other adults in the social group, relative to all other females alive in the population in the same year of life, adjusted for observer effort. These composite agonism indices (AGI) are generated using the same methods as our composite sociality indices but replace grooming with agonisms as the behavior of interest (SCI; discussed below in the Overall Sociality section and in (Archie et al., 2014)). We identified six AGI variables – three that describe agonistic interactions with adult males (M) and three that describe interactions with adult females (F). *AGI (M)* and *AGI (F)* are composite measures that take into consideration all aggressive interactions recorded for the focal individual—including initiating and receiving aggression; *AGI directed (F)* is a composite measure that only take into consideration aggressive interactions that the individual initiated; *AGI received (F)* and *AGI received (M)* are composite measures that only take into consideration aggressive interactions that the individual received from others.

***Category 10: Social dominance rank (blue traits in Table S1).*** We recorded eight different measures of social dominance rank position and change, including: two measures of relative rank, a measure of active rank change, three measures of proportional rank, and two measures of Elo rank.

*Average proportional rank.* Dominance ranks in Amboseli are determined based on the observed outcomes of dyadic aggressive interactions, on a monthly basis (Gordon et al., 2023; Hausfater et al., 1987). Win and loss records are compiled into a pairwise interaction matrix and rank orderings are then assigned to minimize the number of interactions in which lower ranking females won interactions with higher ranking females (Gordon et al., 2023; Hausfater et al., 1987). Proportional rank is defined as the proportion of adult females in an individual’s focal group that the individual outranks, where the lowest-ranking female = 0, and the highest-ranking female = 1. For each female’s adult year of life (from birthdate to birthdate), her proportional rank across the 12 months was averaged.

*Delta proportional rank*. This variable represents the average change in proportional rank throughout each female’s year of life. The variable is calculated by subtracting each month’s proportional rank from the previous month and then averaging the difference throughout the year. A positive delta proportional rank means that the individual rose in rank, on average, and a negative delta proportional rank means that the individual fell in rank, on average.

*Relative delta proportional rank*. This variable represents the average change in proportional rank throughout each female’s year of life, relative to all other females in her group over the same time period. This metric is calculated by subtracting the average delta proportional rank for all individuals in the group from the individual’s average delta proportional rank for the year. A positive value means the individual rose in rank compared to the group average and a negative value means the individual fell in rank compared to the group average.

*Delta Elo rank*. Elo rank is a type of ranking system used in chess and ranked activities, where rank is based on the sequence in which interactions occur and does not require an interaction matrix, like many other rank metrics (Albers & de Vries, 2001; Neumann et al., 2011). Additionally, Elo ranks do not assume equal distance in the hierarchy between consecutively-ranked individuals. For this metric, individuals’ rank scores are updated after each interaction, where the ranking of the winner is increased by an amount that depends on the chance of winning. Thus, if high-ranking individual A wins an interaction with low-ranking individual B (high chance of individual A winning), individual A’s ranking will only increase by a small amount. Conversely, if low-ranking individual B won the interaction (low chance of individual B winning), individual B’s ranking increase would be larger because this outcome indicates that the previous rank order is insufficient to capture observed wins and losses (Albers & de Vries, 2001). While Elo scores can be updated after every individual interaction, we chose to update them once a month, to more closely resemble our ordinal and proportional rank metrics. The variable is calculated by subtracting each individual’s Elo rating in one month from their Elo rating in the previous month and then averaging the difference throughout the year. A positive delta Elo rank means that the individual rose in rank, on average, and a negative delta Elo rank means that the individual fell in rank, on average.

*Relative delta Elo rank*. This variable represents the average change in Elo rank throughout each female’s year of life, relative to all other females in her group over the same time period. This metric is calculated by subtracting the average delta Elo rank for all individuals in the group from the individual’s average delta Elo rank for the year. A positive value means the individual rose in rank compared to the group average and a negative value means the individual fell in rank compared to the group average.

*Rank relative to mother*. Rank relative to mother is a three-level factor that represents whether the individual ranked 1) below her mother, 2) above her mother, or 3) at the same rank as her mother. Female baboons generally follow nepotistic rank ordering, such that female offspring rank immediately below their mother (Hausfater et al., 1987; Lea et al., 2014). However, as individual’s age, they may eventually rise in rank to rank above their mother.

To determine an individual’s rank relative to her mother in a given year of life we considered several different scenarios. If the subject and her mother lived in the same group, we simply compared the ordinal ranks of both individuals and determined whether the subject ranked above or below her mother. We compared their ranks in each month and if the subject ranked above her mother at all in a given year, assigned “ranked above” and if she was below her mother in all months, we assigned “ranked below”. If the subject’s mother was dead, we pulled a list of all individuals in the group that the mother ranked above and below in the month before her death and inspected whether the subject ranked above and below these same individuals. For this assessment, we required that there was at least one common group member shared between the mother in her last month of life and the subject in a given month. We then determined how many times the subject ranked above individuals that previously ranked higher than her mother and how many times the subject ranked below individuals that previously ranked below her mother. If the subject ranked higher than individuals who outranked her mother more times than she ranked below individuals who previously ranked below her mother, she was assigned “ranked above”. Conversely, if she ranked higher than individuals who outranked her mother fewer times than she ranked below individuals who previously ranked below her mother, she was assigned “ranked below”. If the subject did not outrank anyone who outranked her mother or rank below anyone who ranked below her mother, she was assigned “same rank”. If the number of outranked and ranked below individuals were the same, we determined there was not enough information to assign a rank. Similarly, if the individual’s mother was still alive but living in another group, we did not determine her rank relative to her mother. Finally, if none of these scenarios could be investigated, her data were missing and imputed.

*Rank relative to daughter*. Rank relative to daughter is a three-level factor that represents whether the individual 1) did not have any living daughters, 2) ranked below her daughters, or 3) ranked above her daughters. As previously mentioned, daughters often rank immediately below their mothers, but may over time, overtake their mother’s rank (Hausfater et al., 1987; Lea et al., 2014). For all other rank metrics, we used ranking data for adult individuals only; however, for this metric we used a ranking that includes all females (adults and juveniles). Ranks for juveniles are assigned in the same way as adult ranks (using interaction matrices for agonisms; Gordon et al. 2023). If the subject did not yet have any daughters or if she had a daughter who died, she was assigned “no living daughter”. If the subject lived in the same group as one or more of her daughters and she ranked above all of them, she was assigned “ranked above”, but if she ranked below any of them in any month during the year, she was assigned “ranked below”. If the subject lived in a different group from all of her daughters, we determined there was not enough information to assign a relative rank.

*Active rank change.* Active rank change is a three-level factor that represents whether the individual 1) actively rose in rank, 2) actively fell in rank, or 3) experienced no active rank change. Changes in an individual’s rank can occur from active or passive processes. Passive processes include demographic changes, such as births and immigrations, while active processes include all other rank changes, which result from one or more individuals overtaking other individuals in the rank hierarchy (Strauss & Holekamp, 2019). To identify active changes in rank, we constructed a list of all individuals that a subject outranked and was outranked by in each month of life. We then created a similar list in the following month and determined if anyone they previously outranked now outranked them, or if an individual they were previously outranked by was now outranked by them. We summed up the total number of each of these occurrences in each year of life. If, overall, the individual actively rose in rank over more individuals than they fell in rank to, they were assigned “active rise”. Conversely, if the individual actively fell in rank to more individuals than they rose in rank over, they were assigned “active fall”. If the individual did not experience any active rank changes, they were assigned “no active change”. If the individual experienced equal numbers of active rises and falls in rank, we determined there was not enough information to assign them a rank.

2. Measuring covariates of age-related traits

We constructed 78 linear models, one for each female trait of interest (**Table S1**). For each model, we included any variable known or suspected to predict variation in the trait. See **Table S2** for a list of which covariates were included in each model. The data for these variables were collected as follows.

*Age.* Female age was known within a few days’ error for 295 of the females in our data set, and for the remaining 24 females (7.5%), age was known to within 3 months’ error.

*Reproductive state*. Females were assigned to one of three reproductive states—ovarian cycling, pregnancy, or postpartum amenorrhea—using the characteristics described in the “Reproduction” section of the “Measuring age-related traits in female baboons” above. Females experiencing ovarian cycling could be further divided into three phases: follicular, ovulating, and luteal. The follicular phase includes menses and swelling, which is menstruation and the two-week period during which a baboon’s sexual skin fills with fluid. Ovulation occurs most often at the peak of the swelling phase, during the five days prior to the onset of the luteal phase (or deturgescence), when the female’s swelling decreases in size until it is flat. Pregnancy can also be split into three phases, which we refer to as trimesters. The trimesters are represented by 60-day periods, as the average pregnancy duration for female baboons is 180 days (trimester 1 = day 1 to 60, trimester 2 = day 61 to 120, trimester 3 = day 121 to birth).

*Group size.* Group size, as defined here, is a count of the number of adult females in the individual’s social group. Observers record census data during each visit to a study group. Females were classified as adults when they attained menarche (Onyango et al., 2013). For endocrine and parasite traits, group size was recorded on the data of sample collection. For the duration of reproductive states, group size was recorded at the start of the state. For pregnancy outcomes and offspring survival, group size was recorded at the date of conception and offspring birth, respectively. Finally, for activity budgets and maternal care, group size was recorded at the start of each yearly interval (beginning on the individual’s birthdate).

*Daily maximum temperature.* Maximum temperature (in degrees C) was recorded daily using a min-max thermometer located in the ABRP field camp. The time periods over which the mean daily maximum temperature was calculated are as follows: endocrine measures - 1 month before sample collection; cycling duration and pregnancy outcome - 5 months before conception; pregnancy duration - the duration of the pregnancy (i.e., conception up to either birth or fetal loss); postpartum amenorrhea duration and offspring survival - 5 months before offspring birth; parasite measures - 3 months before sample collection; activity budget and maternal care - the duration of time for which the metric was measured.

*Season.* The season (wet vs dry) was determined based on the date of sample collection. The dry season in Amboseli runs from from June to October and the wet season is from November to May.

*Rainfall.* Rainfall (in mm) was recorded daily using a rain gauge located in the ABRP field camp. The time periods over which the average daily rainfall was calculated are as follows: cycling duration and pregnancy outcome - 5 months before conception; pregnancy duration - the duration of the pregnancy; postpartum amenorrhea duration and offspring survival - 5 months before offspring birth; activity budget and maternal care - the duration of time for which the metric was measured.

*Rainfall anomaly.* Rainfall anomaly is a variable that represents how relatively wet or dry a time period is compared to the same time period in previous years. This variable is determined by averaging the rainfall during a given window across all years the Amboseli Baboon Research Project has collected rainfall data (1976–2021 for this analysis), and subtracting this mean from the particular year in question. For the endocrine and parasite traits, rainfall anomaly was always calculated for the three-months prior to sample collection.

*Proportional rank.* The methods for assigning dominance rank are described in the “Rank” section of “Measuring age-associated traits” above and (Gordon et al., 2023; Hausfater et al., 1987). For endocrine and parasite traits, proportional rank was recorded on the day of sample collection. For the duration of reproductive states, proportional rank was recorded at the start of the state. For pregnancy outcomes and offspring survival, proportional rank was recorded on the date of conception and offspring birth, respectively. For overall sociality, dyadic sociality, agonism, and illness and injury traits, proportional rank was recorded at the start of the corresponding year of life. Finally, for activity budgets and maternal care, proportional rank was recorded at the start of each yearly interval (which depends on the presence or absence of an infant and may not directly correspond with the individual’s birthdate).

*Alpha*. Alpha is a two-level factor that represents whether the female was the top-ranking female in her social group. The variable was recorded on the same date that the proportional rank was recorded.

*Storage time as fecal powder.* This variable represents the amount of time that the fecal samples used for steroid hormone analysis were stored as freeze-dried fecal powder at -20^o^C, before being extracted into methanol.

*Storage time in methanol.* This variable represents the amount of time that the fecal samples used for steroid hormone analysis were stored in methanol at -20^o^C, before being assayed.

*Infant sex.* This variable represents the sex of a female’s infant (male or female).

*Habitat quality.* Habitat quality is a two-level binary variable that represents the quality of the habitat at a given time period. In the late-1980s to early-1990s, the ABRP study groups shifted their range ~8 km west, from an area experiencing long-term decline of fever tree groves (*Vachellia xanthophloea*)—which the baboons rely on for sleeping and food—to an area with a relatively high density of fever trees and low density of baboons (Alberts et al., 2005). Before this shift, the habitat is considered “poor” and after this shift, the habitat is considered “good”.

*Maternal presence.* Maternal presence is a two-level binary variable that represents whether the individual’s mother was present in the individual’s social group at the beginning of each year of life.

*Number of adult daughters*. This variable represents the total number of adult daughters that the subject had in her social group at the beginning of each year of life. Daughters are considered adults if they have attained menarche.

3. Measuring early-life adversity

We measured the same six sources of early-life adversity used in Tung *et al.* and several other subsequent papers (Rosenbaum et al., 2020; Tung et al., 2016; Weibel et al., 2020; Zipple et al., 2019). The data underlying these measures were collected as follows.

*Maternal death*. Maternal death occurred if the focal female’s mother died before the focal female reached 4 years of age. Four years represents the earliest age when females attain menarche and become sexually mature (Onyango et al., 2013).

*Presence of a close-in-age younger sibling*. The presence of a close-in-age younger sibling occurred if the focal female’s mother gave birth to another live offspring before the focal female reached 1.5 years of age, which represents the lower quartile of surviving interbirth intervals in this population. A close-in-age younger sibling may have diverted maternal resources from their older sibling early in life.

*Drought*. Drought occurred if the total rainfall during the focal female’s first year of life was less than 200 mm (median annual rainfall is 344 mm). Rainfall is measured daily at the field site using a rain gauge.

*Maternal social isolation*. We calculated maternal social isolation by determining the relative social connectedness of a focal female’s mother to other adult females during the first two years of the focal female’s life. Social connectedness measures were based on a metric of social connectedness (SCI-F) used in previous studies in this population (Tung et al., 2016; Archie et al., 2014). SCI-F measures the mother’s frequency of grooming interactions (both as the actor or recipient) with other adult females in the social group in the same year and is then normalized relative to these rates for all other females alive in the population during the same year. The value was standardized and adjusted for observer effort (Archie et al., 2014; Tung et al., 2016). To transform this measure of social connectedness into a measure of social isolation, we multiplied these values by -1. For the final maternal social isolation variable, negative measures thus represented females with relatively high frequencies of grooming during the designated time period, while positive measures represented females with relatively low frequencies of grooming (i.e., females with socially isolated mothers).

*Low maternal dominance rank*. Females experienced low maternal dominance rank if their mother’s ordinal rank fell into the lowest-ranking quartile for their social group. Maternal dominance rank was defined as the ordinal dominance rank of the female’s mother during the month that the focal female was born. Dominance ranks in Amboseli are determined based on the observed outcomes of dyadic aggressive interactions, on a monthly basis (Altmann, 1974). Win and loss records are compiled into a pairwise interaction matrix and rank orderings are then assigned to minimize the number of interactions in which lower ranking females won interactions with higher ranking females (Gordon et al., 2023; Hausfater et al., 1987).

*Large group size*. Large group size at birth is linked to competition for resources (Rosenbaum et al., 2020; Tung et al., 2016; Weibel et al., 2020; Zipple et al., 2019). Group size was determined based on the total number of adult social group members in the focal female’s social group on the day of her birth. Females were considered to have experienced large group size if their group contained more than 35 adult females/males. Membership in a social group is determined via census data that are collected during regular field observations. Individuals are considered adults if the females have attained menarche and the males have enlarged testes.

4. Comparing regression approaches for building the NPB clock

Before building the final NPB clock, we used the *train* function within the caret package to build three versions of the NPB clock, each based on a different type of regularized regression approach: random forest regression, elastic net regression, and Gaussian process regression (Kuhn, 2022). For each regression model, the predictor variables were the 49 centered and standardized age-associated traits measured annually for each female, and the response variable was each female’s known chronological age. The five imputation sets (described in the main text) were used to predict each female’s age five times, which were averaged to produce her final age prediction for each regression model. We compared the performance of the three methods by evaluating how much variance in predicted age was explained by known chronological age (via R^2^) and the median error in the predicted age estimates for all female years. To understand if females were consistently predicted to be younger or older than their known ages, we calculated the repeatability of “relative NPB age” as the residuals of a linear model regressing predicted against known age. Relative NPB age represents how much older or younger an individual was predicted to be, on average, compared to her true age, with positive values indicating females who were predicted to be older for their known age, and negative values indicating females who were predicted to be younger than their known age.

For the random forest regression, we set the number of trees (ntree) to 2000 and defined the optimal number of variables to randomly sample as candidates at each tree (mtry) as the value that maximized R^2^ between predicted and known age across all samples in the training set. For the elastic net regression, optimal alpha parameters were identified by grid searching across a range of alphas from 0 (equivalent to ridge regression) to 1 (equivalent to Lasso) by increments of 0.1. We defined the optimal alpha as the value that maximized R^2^ between predicted and known age across all samples in the training set. We also set the regularization parameter lambda to the value that maximized R^2^ during 5-fold internal cross-validation. For the Gaussian process regression, we used a radial basis function as our kernel and defined the optimal sigma (the noise standard deviation parameter) as the value that maximized R^2^ between predicted and known age across all samples in the training set. As mentioned in the main text, we used 5-fold cross-validation to generate predicted age estimates for each female in each year of life.

Age predictions from the three supervised machine learning methods (random forest, elastic net regression, and Gaussian process regression) were strongly correlated (R^2^ between all possible pairs of clock predictions was always >0.93; **Table S5**). For each method, the R^2^ for the relationship between predicted and known ages ranged from 0.46 to 0.51, and the median error in predicted age ranged from 2.13 to 2.34 years (**Table S5**). All three methods also produced age predictions that were compressed relative to the 1:1 line, systematically over-predicting the ages of young individuals and under-predicting the ages of old individuals (**Fig. 2**; **Fig. S5**). All methods also found that some females were consistently predicted to be older or younger than their known chronological ages (**Table S5**).

**Fig. S1. Annual physio-behavioral data for adult female baboons.** **(A)** Age in years is shown on the x-axis, and each row on the y-axis represents a unique baboon identity; subjects are ordered from top to bottom by the number of years for which we have data and then by the ages at which those data were collected. A red box surrounds the 25 females with the most years of data represented in **Figure 2B** in the main text. All data are measured across each individual’s year of life (birthdate to birthdate); each point represents a year of life that was included in our machine learning model, with the colors representing the percentage of traits with missing data (before imputation) in that year. The black horizontal lines represent the range of years of data for each female. In total there are data from 2,402 years of life from 319 distinct individuals, with an average of 7.53 years of data per individual. There are some individuals for whom data is missing in early adulthood; most of these individuals were born in the 1970s, at the beginning of the ABRP, before many types of data needed to calculate our traits of interested were collected (e.g., fecal samples, grooming data, agonisms). Therefore, these years of life have too much missing data (see Methods for details on inclusion criteria) and were excluded from the dataset. In addition, almost all 4–5-year-olds have missing data for at least 25% of traits (pink and yellow dots). This occurs because at this age, the females have not yet reproduced and therefore we do not have information for several reproductive and maternal care traits. See also **Table S4** for information on missingness for each trait. **(B)** The inset histogram shows the number of missing traits for each of the 2,402 female years of life in our data set; years missing 32 or fewer traits were included in the NPB clock.

**Fig. S2. The effect of missingness on NPB clock predictions.** Changing the threshold of missingness had little effect on NPB clock performance. The NPB clock in the main text included years that had no more than 32 missing traits (26% missingness for the dataset as a whole). Panel **(A)** shows the baboon’s predicted age in years (Age_P_) from a clock with no more than 18 missing traits per female year (20% missingness for the dataset as a whole) on the y-axis and the baboon’s true chronological age in years (Age_C_) on the x-axis. The dashed line represents the 1:1 relationship between Age_P_ and Age_C_, while the red line shows the linear fit of the model (the R^2^ for the relationship between predicted and known ages was 0.55 with a median error of 2.37 years (compared to R^2^ = 0.51 with a median error of 2.34 for the original clock). Panel **(B)** shows the correlation between the baboon’s predicted age in years (AgeP) generated from the main NPB clock on the x-axis and the revised NPB clock on the y-axis. The red line shows the linear fit of the model. The predicted ages from both clocks are highly correlated (Pearson’s R = 0.96).

**Fig. S3. Forty-nine of the seventy-eight female traits exhibited significant linear or quadratic relationships with female age**. Each plot shows the linear or curvilinear model fit for each trait. Colors indicate the type of trait being modeled (**Fig. 1; Table S1 and S3**). Traits are categorized by the type of variable (continuous traits modeled using a Gaussian error distribution and categorical traits modeled using either binomial or multinomial error distributions). Traits are presented in the order they appear in **Fig. 1B** and **Table S3**; **Table S3** provides standard errors around the estimates.

**Fig. S4. The 49 traits presented in the order they appear in panels Fig 1A-F**. Each plot shows the linear or curvilinear model fit for each trait. Colors indicate the type of trait being modeled (**Fig. 1; Table S1 and S3**). Traits are grouped by the nature of their relationship with age: **(Fig. 1A)** curvilinear concave, **(Fig. 1B)** linear decreasing, **(Fig. 1C)** linear increasing, **(Fig. 1D)** curvilinear plateauing, **(Fig. 1E)** curvilinear ascending, and **(Fig. 1F)** curvilinear concave. Within each group, traits are ordered by the strength of their linear relationship with age (**Table S3**).

**Fig. S5. NPB clocks in wild baboons created using three different machine learning algorithms:** **(A)** random forest regression, **(B)** elastic net regression, and **(C)** Gaussian process regression. The plots show the baboon’s predicted age in years (Age_P_) from the model on the y-axis and the baboon’s true chronological age in years (Age_C_) on the x-axis. The dashed line represents the 1:1 relationship between Age_P_ and Age_C_, while the red line shows the linear fit of the model.

**Fig. S6. Importance plot for the NPB clock** **created using random forests regression**. Because we ran the clock with five different imputations of the data, the importance values for each trait are averaged across the imputation replicates. The standard error is show by the black bars. Traits are colored based on the category that they belong to. Asterisk (*) represent categorical variables.

**Fig. S7. Comparing the NPB clock to other metrics of biological age used in the Amboseli baboon population.** These plots compare the **(A)** R^2^ and **(B)** median error of the NPB clock to seven metrics of biological age. The NPB clock has the third-highest R^2^ (after dentine exposure and the epigenetic clock) and the sixth-lowest median error; however, four of the metrics have a median error within 0.33 years of one another.

**Fig. S8. A variant of the random forest NPB clock with traits that are known predictors of survival excluded (“NPB-restricted”).** The NPB-restricted clock is constructed from 30 variables, excluding known mortality-associated traits: fecal glucocorticoid concentrations (fGC) and all traits that might reflect individual variation in social integration (the incidence of puncture wounds was also excluded due to problems imputing this variable). **(A)** This plot shows the baboon’s predicted age in years (Age_P_) from the model on the y-axis and the baboon’s true chronological age in years (Age_C_) on the x-axis. The dashed line represents the 1:1 relationship between Age_P_ and Age_C_, while the red line shows the linear fit of the model. **(B)** This plot shows the correlation between the baboon’s predicted age in years (AgeP) generated from the main NPB clock on the x-axis and the NPB-restricted clock on the y-axis. The predicted ages from both clocks are highly correlated (Pearson’s R = 0.93).

Lifetime relative

NPB-restricted age

Annual relative

NPB-restricted age

**Fig. S9. Individuals who look oldest for their age live shorter lives, excluding known mortality-associated traits from the main NPB clock.** These plots show female survival as a function of **(A)** her mean lifetime Age_Δ_, or (**B**) Age_Δ_ in each year of life, when Age_Δ_ is calculated from the NPB-restricted clock (which excludes all known mortality-associated traits). Within each plot the four curves represent each quartile of the delta age estimate, with quartile 1 (blue) representing individuals who look youngest for their age and quartile 4 (pink) representing individuals who look oldest for their age.

**Fig. S10. Individual sources of adversity are not linked to biological age predictions.** This plot shows the residuals for delta age (Age_Δ_), controlling for chronological age (Age_C_) and three social/environmental variables in adulthood (proportional dominance rank, rainfall anomaly, and social group size) as a function of each of the individual sources of adversity on the x-axis: **(A)** maternal rank (B=0.03, P=0.10), **(B)** group size (B=-0.01, P=0.29), **(C)** maternal social isolation (B=-0.13, P=0.13), **(D)** maternal death (B=0.17, P=0.22), **(E)** close-in-age sibling (B=0.08, P=0.61), and **(F)** drought (B=0.14, P=0.43. No individual source of adversity significantly predicted biological age.

**REFERENCES**

Akinyi, M. Y., Jansen, D., Habig, B., Gesquiere, L. R., Alberts, S. C., & Archie, E. A. (2019). Costs and drivers of helminth parasite infection in wild female baboons. *Journal of Animal Ecology*. https://doi.org/10.1111/1365-2656.12994

Albers, P. C. H., & de Vries, H. (2001). Elo-rating as a tool in the sequential estimation of dominance strengths. *Animal Behaviour*, *61*(2), 489–495. https://doi.org/10.1006/anbe.2000.1571

Alberts, S., Archie, E., Altmann, J., & Tung, J. (2020). *Monitoring guide for the Amboseli Baboon Research Project*. https://amboselibaboons.nd.edu/assets/384683/abrp_monitoring_guide_9april2020.pdf

Alberts, S. C., Hollister-Smith, J. A., Mututua, R. S., Sayialel, S. N., Muruthi, P. M., Warutere, J. K., & Altmann, J. (2005). Seasonality and long-term change in a savannah environment. In C. P. van Schaik & D. K. Brockman (Eds.), *Seasonality in Primates: Studies of Living and Extinct Human and Non-Human Primates* (pp. 157–195). Cambridge University Press.

Altmann, J. (1974). Observational study of behavior: Sampling methods. *Behaviour*, *49*(3/4), 227–267.

Altmann, S. A. (1973). The Pregnancy Sign in Savannah Baboons. *The Journal of Zoo Animal Medicine*, *4*(2), 8–12.

Altmann, S. A. (1998). *Foraging for survival: Yearling baboons in Africa.* University of Chicago Press.

Archie, E. A., Tung, J., Clark, M., Altmann, J., & Alberts, S. C. (2014). Social affiliation matters: Both same-sex and opposite-sex relationships predict survival in wild female baboons. *Proceedings of the Royal Society B*, *281*, 1–9.

Cheney, D. L., Silk, J. B., & Seyfarth, R. M. (2016). Network connections, dyadic bonds and fitness in wild female baboons. *Royal Society Open Science*, *3*(7), 160255. https://doi.org/10.1098/rsos.160255

Gesquiere, L. R., Altmann, J., Khan, M. Z., Couret, J., Yu, J. C., Endres, C. S., Lynch, J. W., Ogola, P., Fox, E. A., Alberts, S. C., & Wango, E. O. (2005). Coming of age: Steroid hormones of wild immature baboons (Papio cynocephalus). *American Journal of Primatology*, *67*(1), 83–100. https://doi.org/10.1002/ajp.20171

Gesquiere, L. R., Khan, M., Shek, L., Wango, T. L., Wango, E. O., Alberts, S. C., & Altmann, J. (2008). Coping with a challenging environment: Effects of seasonal variability and reproductive status on glucocorticoid concentrations of female baboons (Papio cynocephalus). *Hormones and Behavior*, *54*(3), 410–416. https://doi.org/10.1016/j.yhbeh.2008.04.007

Gesquiere, L. R., Pugh, M., Alberts, S. C., & Markham, A. C. (2018). Estimation of energetic condition in wild baboons using fecal thyroid hormone determination. *Gen Comp Endocrinol*, *260*, 9–17. https://doi.org/10.1016/j.ygcen.2018.02.004

Gesquiere, L. R., Wango, E. O., Alberts, S. C., & Altmann, J. (2007). Mechanisms of sexual selection: Sexual swellings and estrogen concentrations as fertility indicators and cues for male consort decisions in wild baboons. *Hormones and Behavior*, *51*(1), 114–125. https://doi.org/10.1016/j.yhbeh.2006.08.010

Gordon, J., Jansen, D., Learn, N., Altmann, J., Tung, J., Archie, E., & Alberts, S. (2023). *Ordinal dominance rank assignments: Protocol for the Amboseli Baboon Research Project*. https://amboselibaboons.nd.edu/assets/509243/gordon_etal_elo_matrix_rank_comparisons_22mar2023_for_abrp_website_google_docs.pdf

Habig, B., Jansen, D. A. W. A. M., Akinyi, M. Y., Gesquiere, L. R., Alberts, S. C., & Archie, E. A. (2019). Multi-scale predictors of parasite risk in wild male savanna baboons (Papio cynocephalus). *Behavioral Ecology and Sociobiology*, *73*(10), 134. https://doi.org/10.1007/s00265-019-2748-y

Hausfater, G., Cairns, S. J., & Levin, R. N. (1987). Variability and Stability in the Rank Relations of Nonhuman Primate Females: Analysis by Computer Simulation. *American Journal of Primatology*, *12*, 55–70.

Khan, M. Z., Altmann, J., Isani, S. S., & Yu, J. (2002). A matter of time: Evaluating storage of fecal samples for steroid analysis. *General and Comparative Endocrinology*, *128*, 57–64.

Lea, A. J., Learn, N. K., Theus, M. J., Altmann, J., & Alberts, S. C. (2014). Complex sources of variance in female dominance rank in nepotistic society. *Animal Behaviour*, *94*, 87–99.

Lynch, J. W., Khan, M. Z., Altmann, J., Njahira, M. N., & Rubenstein, N. (2003). Concentrations of four fecal steroids in wild baboons: Short-term storage conditions and consequences for data interpretation. *General and Comparative Endocrinology*, *132*(2), 264–271. https://doi.org/10.1016/S0016-6480(03)00093-5

Neumann, C., Duboscq, J., Dubuc, C., Ginting, A., Irwan, A. M., Agil, M., Widdig, A., & Engelhardt, A. (2011). Assessing dominance hierarchies: Validation and advantages of progressive evaluation with Elo-rating. *Animal Behaviour*, *82*(4), 911–921. https://doi.org/10.1016/j.anbehav.2011.07.016

Onyango, P. O., Gesquiere, L. R., Altmann, J., & Alberts, S. C. (2013). Puberty and dispersal in a wild primate population. *Hormones and Behavior*, *64*, 240–249.

Rosenbaum, S., Zeng, S., Campos, F. A., Gesquiere, L. R., Altmann, J., Alberts, S. C., Li, F., & Archie, E. A. (2020). Social bonds do not mediate the relationship between early adversity and adult glucocorticoids in wild baboons. *Proceedings of the National Academy of Sciences*, 202004524. https://doi.org/10.1073/pnas.2004524117

Shaikh, A., Shaikh, S., Celaya, C., & Gomez, I. (1982). Temporal relationship of hormonal peaks to ovulation and sex skin deturgescence in the baboon. *Primates*, *23*, 444–452.

Strauss, E. D., & Holekamp, K. E. (2019). Social alliances improve rank and fitness in convention-based societies. *Proceedings of the National Academy of Sciences*, *116*(18), 8919–8924. https://doi.org/10.1073/pnas.1810384116

Tung, J., Archie, E. A., Altmann, J., & Alberts, S. C. (2016). Cumulative early life adversity predicts longevity in wild baboons. *Nat Commun*, *7*, 11181. https://doi.org/10.1038/ncomms11181

Weibel, C. J., Tung, J., Alberts, S. C., & Archie, E. A. (2020). Accelerated reproduction is not an adaptive response to early-life adversity in wild baboons. *Proceedings of the National Academy of Sciences*, *117*(40), 24909–24919. https://doi.org/10.1073/pnas.2004018117

Wildt, D., Doyle, L., Stone, S., & Harrison, R. (1977). Correlation of perineal swelling with serum ovarian hormone levels, vaginal cytology, and ovarian follicular development during the baboon reproductive cycle. *Primates*, *18*, 261–270.

Zipple, M. N., Archie, E. A., Tung, J., Altmann, J., & Alberts, S. C. (2019). Intergenerational effects of early adversity on survival in wild baboons. *eLife*, *8*(e47433).
